# Effects of Maternal Empowerment on Childhood Undernutrition in Bangladesh: Findings from Nationally Representative Surveys

**DOI:** 10.3390/nu18111730

**Published:** 2026-05-28

**Authors:** M. A. Rifat, Rokibul Islam, Rinath Bintey Didar, Syeda Saima Alam, Sania Nusrat Urmee, Joya Bhowmick, Plabon Sarkar, Md. Ruhul Amin, Sanjib Saha

**Affiliations:** 1 BRAC Institute of Educational Development, BRAC University, Dhaka 1212, Bangladesh; 2 University of Dhaka, Dhaka 1000, Bangladesh; rirokib6@gmail.com (R.I.); ruhul.infs@du.ac.bd (M.R.A.); 3 Noakhali Science and Technology University, Noakhali 3814, Bangladesh; 4 Department of Food and Nutrition, National College of Home Economics, Dhaka 1207, Bangladesh; joyabhowmick62@gmail.com; 5 Department of Clinical Science (Malmö), Lund University, 22362 Lund, Sweden

**Keywords:** maternal empowerment, child nutrition, undernutrition, BDHS, Bangladesh

## Abstract

Background/Objectives: Empowered mothers are more likely to adopt recommended childcare practices, thereby contributing to reduced childhood undernutrition. However, the magnitude of the association between maternal empowerment and childhood undernutrition in Bangladesh has not been comprehensively assessed. This study aims to address this research gap. Methods: The Bangladesh Demographic and Health Survey (BDHS) 2017-18 and BDHS 2022 served as data sources. Maternal empowerment was assessed across three domains, e.g., attitude to violence, social independence, and decision making, using the Survey-based Women’s Empowerment (SWPER) index. The undernutrition status of children was assessed through z-score based indicators, including stunting (height-for-age z-score < −2 SD), wasting (weight-for-height z-score < −2 SD), and underweight (weight-for-age < −2 SD). Children with at least two and any of these undernutrition conditions were categorized as multiple undernutrition and any undernutrition, respectively. Multivariable logistic regression models were utilized to observe the survey-specific and pooled association between maternal empowerment and childhood undernutrition. Results: The analysis includes 11,647 mother–child pairs. The association between maternal empowerment and childhood undernutrition was consistent across individual surveys and the pooled sample, although the significance level varied by empowerment domains and undernutrition categories. Maternal social independence was found to be a significant protective factor against both multiple and any childhood undernutrition status in individual surveys and the pooled sample. For example, in the pooled sample, high maternal empowerment in the social independence domain was significantly associated with 18% (AOR: 0.82; 95% CI: 0.69, 0.98; *p* = 0.026) lower odds of multiple undernutrition statuses and 18% (AOR: 0.82; 95% CI: 0.71, 0.95; *p* = 0.009) lower odds of any undernutrition statuses than those of low maternal empowerment. Conclusions: Improving the status of maternal social independence can potentially result in reduced childhood undernutrition. The scope remains to cascade the benefits of the other two maternal empowerment domains, e.g., attitude to violence and decision making, to child nutrition in Bangladesh.

## 1. Introduction

Childhood undernutrition is a physiological condition primarily caused by inadequate supply of macro- and micronutrients in the body and generally assessed as stunting (low height-for-age), wasting (low weight-for-height), underweight (low weight-for-age), and micronutrient deficiencies [1]. In low- and middle-income countries, childhood undernutrition is a major public health challenge, resulting in compromised immunity, prolonged developmental delay, frequent episodes of morbidity, and elevated risks of mortality among children [2,3,4]. Globally, one in every five children under five years of age is suffering from chronic undernutrition (stunting), and approximately six percent of children are suffering from acute undernutrition (wasting) [5]. According to UNICEF-WHO-World Bank Joint Child Malnutrition Estimates 2025, South Asia hosts a significant proportion of global undernourished children, representing 31.4% and 13.6% prevalence of childhood stunting and wasting, respectively [5]. Although the prevalence of childhood undernutrition in Bangladesh has been significantly reduced in recent decades, the scenario is still worse than global estimates. According to the latest Bangladesh Demographic and Health Survey (BDHS 2022), 24%, 11%, and 22% of children 6–59 months of age were assessed as stunted, wasted, and underweight, respectively [6].

Childhood undernutrition is a consequence catalyzed by a wide range of factors broadly categorized as immediate, underlying, and enabling determinants. Some of these factors include inadequate dietary intake, infection, household food insecurity, suboptimum care practices, poor hygiene and sanitation situation, low socio-economic condition, and fragile governance [7,8]. Therefore, addressing childhood undernutrition requires multifaceted and multidimensional approaches [9,10]. Mothers play the role of primary care providers to their children and make decisions regarding infant and young child feeding; therefore, maternal empowerment stands for a significant determinant of child health and nutrition, especially in low- and middle-income settings [11,12,13]. Previous study revealed that educated mothers have greater access to better choices of food and knowledge on recommended feeding practices, which are essential for child nutrition [14]. Furthermore, the potential for income generation enables mothers to have more influence over family budgets for food purchases and intra-household food distribution. This has a positive impact on recommended complementary feeding practices for children [15]. Mothers who have decision-making capacity and health literacy are better equipped to navigate healthcare systems, ensuring timely care for themselves and their children, advocating for essential services, accessing vaccinations, and participating in maternal and child health programs [16,17].

Maternal empowerment refers to a process by which mothers acquire the ability to make strategic life choices [18]. It is a multidimensional construct, covering a broad spectrum of social, economic, political, and psychological aspects, which are associated with their autonomy, mobility, decision-making capacity, and control over resources [19]. For example, an empowered mother is likely to have better knowledge and control over resources, which she can efficiently utilize to ensure the recommended care practices for her child. Therefore, assessing the status of maternal empowerment might appear challenging. A systematic literature review revealed that methods to assess women’s agency, i.e., decision-making capacity and freedom of movement, varied across studies, resulting in different degrees of associations between women’s agency and their healthcare practices in lower- and middle-income countries [20]. Hence, a tool named Survey-based Women’s Empowerment Index (SWPER) is validated for nationally representative surveys conducted in low- and middle-income countries under the Demographic and Health Survey (DHS) program [21,22,23,24,25].

Studies conducted in other settings, including Ethiopia, Kenya, Rwanda, Tanzania, and Uganda, identified a positive association between maternal empowerment and child nutrition among the lowest wealth category families [26]. In Bangladesh, maternal empowerment, as estimated using the SWPER index, was found to be positively associated with their utilization of care from pregnancy to the postpartum period [27,28]. It is therefore reasonable to hypothesize that high levels of maternal empowerment contribute to a reduction in childhood undernutrition. However, the magnitude of association remained unexamined in the Bangladeshi context. Furthermore, studies investigating the factors associated with the nutritional status of children in Bangladesh and South Asia primarily considered maternal socioeconomic characteristics, e.g., education and occupation, in their analysis, rather than examining the multidimensional constructs of maternal empowerment as considered in the SWPER index [29,30,31,32]. Therefore, the current study aims to fill this research gap through quantifying the relationship between maternal empowerment and childhood undernutrition in Bangladesh, utilizing the publicly available and high-quality data from two consecutive nationally representative surveys conducted within the last ten-year period. Findings can serve as evidence for policymakers and non-government organizations to comprehend the relationship between maternal empowerment and childhood undernutrition and identify strategies to translate the benefits of maternal empowerment in preventing childhood undernutrition in Bangladesh.

## 2. Materials and Methods

### 2.1. Study Design and Sample

Two cross-sectional nationally representative surveys, i.e., BDHS 2017-18 and BDHS 2022, served as datasets in this study [6,33]. These surveys were designed following a two-stage stratified cluster random sampling technique and had a response rate of more than 98%. Both surveys had a sample size representative of the urban and rural populations of Bangladesh. In order to ensure national representativeness, the survey clusters include geographical areas such as river basins, hills, coastal areas, islands, and plain land across the country. The methods of the surveys are detailed elsewhere [6,33].

**Figure 1:**
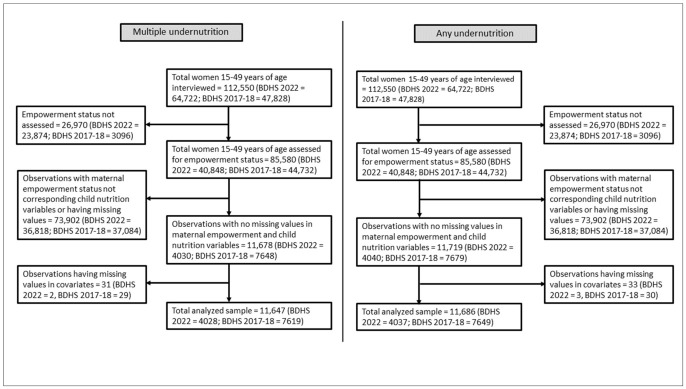
Selection process of the analyzed sample from the Bangladesh Demographic and Health Survey (BDHS) 2022 and BDHS 2017-18.

The study participants are children 6–59 months (<5 years) of age and their mothers 15–49 years of age from eight divisions in Bangladesh, representing both urban and rural areas. To obtain the analyzed sample, observations corresponding to maternal empowerment-related variables, childhood undernutrition-related variables, and covariates were merged in individual survey datasets. Finally, these individual survey round datasets were appended to obtain the final analyzed sample. The analysis was conducted with observations containing no missing values in women empowerment indicators, childhood undernutrition indicators, and considered covariates. The selection process of the analyzed sample is portrayed in Figure 1. In this study, the Strengthening the Reporting of Observational studies in Epidemiology (STROBE) was considered as a reporting guideline [34]. The STROBE checklist is provided as the supplementary file (Supplementary Material Table S1).

### 2.2. Exposure Variables

The exposure variables are the maternal empowerment measured by the Survey-based Women’s Empowerment Index (SWPER), an index that estimates empowerment status across three domains [21,22]. These are (1) the attitudes to violence domain which is closely related to the concept of intrinsic agency, serving as a proxy for the woman’s incorporation of gender norms concerning the acceptability of violence (perceived justification of beating wife in situation such as going out without telling husband, neglecting child, arguing with husband, refusing to have sex with husband, and burning foods), (2) the social independence domain which is mainly composed of preconditions that enable women to achieve their goals (schooling attainment, capacity to access information such as reading newspaper, age at pivotal life events such as first cohabitation and first birth, age difference with husband, and difference in educational attainment with husband), and (3) the decision making domain that is the extent of women’s participation in the household decision making process (such as who decides on healthcare, who decides on large household purchase, and who decides on visits to family or relatives) [21]. These domains are generated as a continuous standardized score using principal component analysis. Therefore, in each of the three domains, the status of empowerment was categorized into three, including low empowerment, medium empowerment, and high empowerment. Items constructing the SWPER index and the cutoffs for categorization are provided in the Supplementary Material Table S2.

### 2.3. Outcome Variables

The outcome variables are the undernutrition status of the children, including multiple undernutrition and any undernutrition. These estimates were calculated based on the status of stunting (low height-for-age z-score or HAZ < −2 SD), underweight (low weight-for-age z-score or WAZ < −2 SD), and wasting (low weight-for-height z-score or WHZ < −2 SD) among children 6–59 months of age [1,35,36]. To assess the undernutrition status of sampled children, body weight, height (for children aged 2–5 years) and length (for children aged < 2 years), edema, and age were measured following World Health Organization (WHO) standards [37]. These measures were then compared with the reference population of the WHO growth standard 2006, specific for boys and girls, to obtain the individual z-scores of sampled children [37]. A child was considered to have multiple undernutrition if the child had at least two forms of undernutrition, i.e., stunting and underweight, stunting and wasting, wasting and underweight, and all three forms. A child was assessed as having any undernutrition if the child had any form of undernutrition. Outcome variables are binary; therefore, children having a status of multiple undernutrition and any undernutrition were coded as 1, or otherwise as 0.

### 2.4. Covariates

Covariates were selected based on their expected association with exposure and outcome variables and evidence from the published literature [38,39,40]. Therefore, the following covariates were considered: education level of children’s father (no education, primary, secondary, and higher), occupation status of children’s mother (not working, working), occupation status of children’s father (not working, working), wealth index (poorest, poorer, middle, richer, richest), mothers’ status facing problem while accessing healthcare (not big problem, big problem), maternal body mass index (underweight, normal, overweight, obese), religion (Muslim, other), place of residence (rural, urban), division of residence (Barisal, Chittagong, Dhaka, Khulna, Mymensingh, Rajshahi, Rangpur, Sylhet), and survey round (BDHS 2017-18, BDHS 2022). Maternal status of facing a problem while accessing healthcare was composed of three variables: (1) problem while seeking permission, (2) problem while accessing money, and (3) problem due to distance from the health facility. If a mother perceived any of these three as a big problem during accessing healthcare, the mother was considered to face a big problem and subsequently categorized under “big problem”, or otherwise “not big problem”, in the analyzed dataset. Selection of covariates was conducted in such a way that they do not overlap with any items included in the SWPER index.

### 2.5. Statistical Analysis

Cross-tabulation was used to observe the distribution of the status of childhood undernutrition domains (multiple undernutrition and any undernutrition) by maternal empowerment domains and covariates. The differences in distribution of maternal empowerment status and covariates by status of childhood undernutrition were observed using Chi-squared tests. The association between childhood undernutrition and maternal empowerment was estimated by multivariable logistic regression models. Logistic regression models were constructed to individually estimate the association between each of the maternal empowerment domains and the status of childhood undernutrition, including multiple and any undernutrition. As the surveys employed a stratified cluster random sampling design, the models were adjusted for primary sampling unit, sampling strata, sampling weight, and covariates. In the pooled data, weight was created using unique primary sampling units and strata created from individual datasets. Furthermore, to adjust survey-specific effects, the year of survey was considered as a covariate while conducting pooled data analysis [41]. Adjusted odds ratios (AORs) were calculated to observe the effect, considering maternal low empowerment status as the reference category.

Multicollinearity was observed through estimating the Variance Inflation Factor (VIF), considering VIF < 5 as an acceptable level of multicollinearity [42]. All the analyses were conducted considering *p* < 0.05 as statistically significant. STATA software version 17 (StataCorp, College Station, TX, USA) was used to conduct statistical analysis. Univariable and multivariable regression models were run using the “svy” command.

## 3. Results

### 3.1. Sample Characteristics and Distribution of Exposures and Outcomes

**Figure 2:**
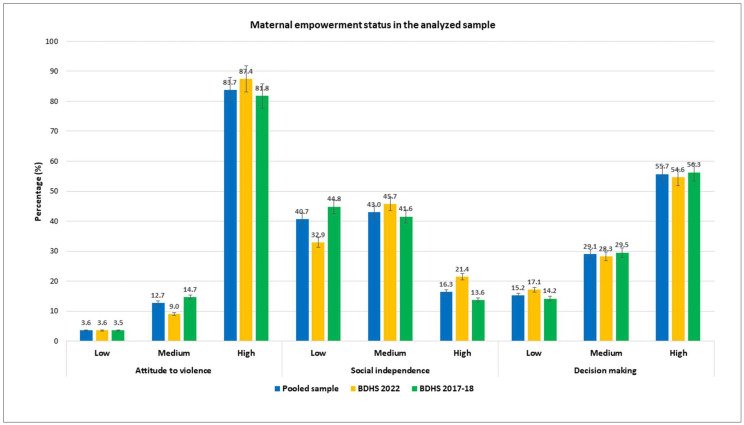
Status of maternal empowerment in Bangladesh Demographic and Health Survey (BDHS) 2022, BDHS 2017-18, and pooled sample.

A total of 11,686 mother–child pairs (BDHS 2022 = 4037 and BDHS 2017-18 = 7649) were included in the analysis. The majority of the mothers in the pooled sample and individual surveys were highly empowered concerning the attitude to violence and decision-making domains. In contrast, in the social independence domain, the proportion of highly empowered mothers was lower than that of low- and medium-empowered mothers in pooled and individual survey samples (Figure 2).

**Figure 3:**
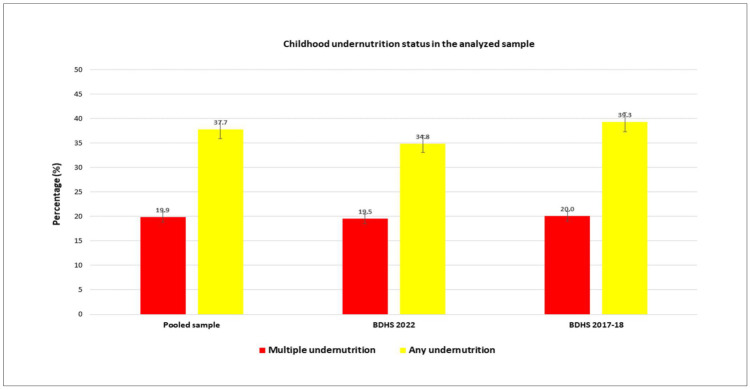
Status of multiple undernutrition and any undernutrition among children in the Bangladesh Demographic and Health Survey (BDHS) 2022, BDHS 2017-18, and pooled sample.

On the other hand, proportions of children 6–59 months of age with multiple and any forms of undernutrition in the pooled sample were 19.87% and 37.53%, respectively (Figure 3).

The distribution of multiple and any forms of childhood undernutrition by domains of maternal empowerment and covariates in BDHS 2022, BDHS 2017-18, and the pooled sample is presented in Table 1. The proportion of children 6–59 months of age with multiple forms of undernutrition significantly differed by attitude to violence and social independence domains of maternal empowerment, fathers’ education, wealth index, maternal BMI, type of place of residence, and division. Prevalence of multiple forms of childhood undernutrition significantly differed by maternal occupation and accessing healthcare in the BDHS 2017-18 and the pooled sample but not in BDHS 2022. Furthermore, the proportion of children with any form of undernutrition was significantly varied by the social independence domain of maternal empowerment, fathers’ education, wealth index, maternal BMI, type of place of residence, and division in pooled and individual survey samples. The distribution of any childhood undernutrition significantly differed by maternal attitude to violence and maternal occupation in the BDHS 2017-18 and the pooled sample but not in the BDHS 2022. The distribution of any childhood undernutrition significantly differed by year of survey; however, such a difference was not observed for multiple childhood undernutrition.

### 3.2. Effects of Maternal Empowerment on Multiple Childhood Undernutrition

The adjusted association between the status of multiple forms of childhood undernutrition and the maternal empowerment domains in pooled and individual survey samples is presented in Table 2. In the pooled sample, the multivariable logistic regression revealed that medium and high maternal empowerment in the social independence domain were associated with 19% (AOR: 0.81; 95% CI: 0.73, 0.91; *p* < 0.001) and 18% (AOR: 0.82; 95% CI: 0.69, 0.98; *p* = 0.026) lower odds of multiple forms of undernutrition among children than those of low maternal empowerment, respectively. Additionally, high maternal empowerment in the attitude to violence domain was associated with 30% (AOR: 0.70; 95% CI: 0.54, 0.90; *p* = 0.007) lower odds of multiple undernutrition status than that of low maternal empowerment. In BDHS 2022, medium and high maternal empowerment in the social independence domain was associated with 27% (AOR: 0.73; 95% CI: 0.59, 0.89; *p* = 0.002) and 25% (AOR: 0.75; 95% CI: 0.57, 0.99; *p* = 0.041) lower odds of multiple childhood undernutrition than those of low maternal empowerment, respectively. Additionally, high maternal empowerment was associated with 37% (AOR: 0.63; 95% CI: 0.41, 0.97; *p* = 0.034) lower odds of multiple childhood undernutrition than that of low empowerment in the attitude to violence domain. In BDHS 2017-18, only the social independence domain was found to be significantly associated with multiple forms of childhood undernutrition, demonstrating that medium maternal empowerment reduces the odds of multiple childhood undernutrition by 14% (AOR: 0.86; 95% CI: 0.75, 0.98; *p* = 0.024) compared to that of low maternal empowerment. No significant association was found between the decision making domain of maternal empowerment and multiple undernutrition status among children in either the pooled sample or individual surveys.

### 3.3. Effects of Maternal Empowerment on Any Childhood Undernutrition

The association between maternal empowerment domains and the status of any form of childhood undernutrition is presented in Table 3. The multivariable logistic regression analysis revealed that medium and high maternal empowerment in the social independence domain was associated with 13% (AOR: 0.87; 95% CI: 0.79, 0.96; *p* = 0.007) and 18% (AOR: 0.82; 95% CI: 0.71, 0.95; *p* = 0.009) lower odds of any undernutrition status among children, respectively, compared to those of low maternal empowerment in the pooled sample. In BDHS 2022, medium maternal empowerment in the social independence domain was associated with 23% (AOR: 0.77; 95% CI: 0.65, 0.90; *p* = 0.002) lower odds of any childhood undernutrition status compared to those of low maternal empowerment. High maternal empowerment in the social independence domain exhibited a significant association with 22% (AOR: 0.78; 95% CI: 0.64, 0.95; *p* = 0.014) lower odds of any undernutrition status of children in BDHS 2017-18. In adjusted models, no significant association was observed between the status of any childhood undernutrition and the attitude to violence and decision making domains of maternal empowerment in the pooled sample and individual surveys.

Effects of maternal empowerment domains on stunting, wasting, and underweight are presented in the supplementary file (Supplementary Material Tables S3–S5). The analysis revealed that mothers with medium and high empowerment status in the social independence domain were significantly associated with lower odds of childhood stunting and underweight in the BDHS 2022 and pooled sample. Detailed multivariable regression models showing the effects of maternal empowerment domains on any and multiple childhood undernutrition are presented in supplementary file (Supplemental Material Tables S6–S11). In the adjusted models, no multicollinearity across the covariates was detected.

## 4. Discussion

In this study, we have identified different degrees of association between childhood undernutrition categories and maternal empowerment domains in individual surveys and the pooled sample. In all cases, the social independence domain of maternal empowerment appeared to be a protective factor for children from being undernourished, demonstrating a significant association with both multiple and any forms of undernutrition in Bangladesh. The adjusted effect of the maternal attitude to violence domain on multiple forms of childhood undernutrition demonstrated a significant association in BDHS 2022 and pooled samples; however, its effect on any childhood undernutrition was not significant either in individual surveys or in the pooled sample. On the other hand, no statistically significant association between childhood undernutrition categories and the decision making domain of maternal empowerment was identified in any of the analyzed datasets.

Our findings demonstrate that the social independence domain had a stronger effect on the low likelihoods of multiple and any forms of childhood undernutrition than the other two maternal empowerment domains, i.e., attitude to violence and decision making. Such differences could be because of the nature of the items constructed in these maternal empowerment domains. For example, items such as maternal educational attainment and frequency of reading newspapers and magazines were significantly associated with childhood undernutrition (Supplementary Material Table S12). For example, the social independence domain consists of variables such as educational attainment and frequency of reading newspapers or magazines, which are individually associated with the nutritional status of children [39,43,44]. Evidence showed that high maternal educational attainment and delivery at adulthood were associated with better care practices, which contribute to better child nutrition [43,45]. A multi-country study revealed that maternal education and control over reproduction corresponded to improved child nutrition in developing countries [46]. Furthermore, the frequency of reading newspapers increases maternal potential to access information about child health and nutrition from the mass media [47].

There are several possible explanations for why the decision making domain of maternal empowerment did not exhibit any significant association with childhood undernutrition in our analysis. This domain includes items which may not directly translate into reduced childhood undernutrition. For example, large household purchases vary by household socioeconomic status and may not guarantee health- or food-related expenditures [48,49]. Similarly, visiting relatives does not necessarily confirm maternal support or transfer of knowledge related to child feeding or care practices. These nuances, however, warrant further investigation. On the other hand, the attitude to violence domain, which reflects maternal intrinsic agency, includes items such as perceived justification of spousal violence for neglecting children or burning food—situations that may relate to childhood undernutrition. Other items in this domain, however, e.g., related to going out without permission, arguing with husband, and refusing to have sex, are more indicative of the power dynamics in the marital relationship and may have a weaker or indirect association with childhood undernutrition status. These hypotheses also require further investigation.

The analyzed sample size for multiple undernutrition categories was slightly smaller than that of any undernutrition category. This was because the conditions to assess multiple undernutrition are stricter than those to assess any undernutrition status. In this study, both BDHS 2017-18 and BDHS 2022 were included. The decision to include the latest two nationally representative surveys was to synthesize updated evidence covering a broad period of time, especially after the implementation of the national nutrition policy of Bangladesh in 2015 [50].

Although methods used to assess maternal empowerment varied across studies, our findings show consistency with the synthesis of previous research conducted in diverse settings. In Bangladesh, enhancing maternal empowerment has been recommended to uplift the child nutrition situation [51]. Another study found maternal empowerment had a significant relationship with child health, especially with low stunting, wasting, and underweight in Asian developing countries [52]. A systematic review conducted from a South Asian perspective unveiled different degrees of association between maternal empowerment domains and child anthropometry, concluding a positive relationship between maternal empowerment and child nutrition [53]. Findings from Cameroon, Benin, and India also exhibited a positive association between maternal empowerment and child nutrition [54,55,56].

### Limitations and Strengths

This study has several limitations. The cross-sectional nature of the surveys limits the scope to infer causal association between maternal empowerment and child nutrition. Although the SWPER index is a validated tool comprising pertinent items to measure maternal empowerment, it does not fully capture its multidimensional aspects, e.g., economic, political, psychological, and socio-cultural aspects [57,58]. Furthermore, estimation of empowerment domains, such as attitude to violence and decision making, includes items that are highly related to maternal perception and sensitive to social desirability bias. Another limitation is that the undernutrition status in this study was assessed only through indicators based on anthropometric assessment, excluding micronutrient deficiencies, which are also important undernutrition indicators. Despite the aforementioned limitations, a few strengths are also worth mentioning. Utilization of nationally representative data provides high statistical power. Combining data from two surveys allows us to generalize the findings for a broader period of time and observe differential effects across survey rounds. Consideration of any and multiple forms of undernutrition offers a more holistic view than considering individual undernutrition categories such as stunting, wasting, and underweight, which are distinct in nature, considering their physiology and prognosis. In the Bangladesh context, this is the first study unfolding how maternal empowerment domains contribute to child nutrition.

Findings can be useful in making evidence-based policies, strategies, and interventions to improve maternal empowerment so that the benefits can be translated to the better nutritional status of children in Bangladesh.

## 5. Conclusions

In Bangladesh, maternal empowerment appeared to be protective against childhood undernutrition, although the degrees of association vary depending on maternal empowerment domains and childhood undernutrition categories. A high level of maternal empowerment in the social independence domain has the potential to prevent childhood undernutrition by 18%, which was observed in both surveys and the pooled sample. However, the association was significant for the maternal social independence domain, implying that an increased level of maternal social independence can potentially reduce childhood undernutrition in Bangladesh. On the other hand, cascading the benefits of the other two maternal empowerment domains, e.g., attitude to violence and decision making, to child nutrition still remains as a scope in Bangladesh. Instead of evidence synthesized from cross-sectional surveys, future studies may consider a longitudinal follow-up design to estimate causality and compare with current findings.

## Figures and Tables

**Table 1:** Distribution of the statuses of childhood undernutrition by maternal empowerment and covariates in the analyzed sample.

Variables	Multiple Undernutrition			Any Undernutrition		
	Pooled (n = 11,647)	BDHS 2022 (n = 4028)	BDHS 2017-18 (n = 7619)	Pooled (n = 11,686)	BDHS 2022 (n = 4037)	BDHS 2017-18 (n = 7649)
Attitude to violence	[*p* < 0.001]	[*p* = 0.005]	[*p* = 0.008]	[*p* = 0.003]	[*p* = 0.075]	[*p* = 0.083]
Low empowerment	112 (27.25)	42 (29.58)	70 (26.02)	175 (42.17)	59 (40.97)	116 (42.80)
Medium empowerment	322 (21.68)	77 (21.15)	245 (21.86)	608 (40.89)	140 (38.46)	468 (41.67)
High empowerment	1880 (19.28)	668 (18.97)	1212 (19.46)	3627 (37.07)	1206 (34.17)	2421 (38.71)
Social independence	[*p* < 0.001]	[*p* < 0.001]	[*p* < 0.001]	[*p* < 0.001]	[*p* < 0.001]	[*p* < 0.001]
Low empowerment	1113 (23.53)	318 (24.09)	795 (23.31)	2020 (42.49)	533 (40.20)	1487 (43.38)
Medium empowerment	930 (18.56)	341 (18.49)	589 (18.59)	1837 (36.56)	611 (33.10)	1226 (38.58)
High empowerment	271 (14.23)	128 (14.81)	143 (13.75)	553 (28.98)	261 (30.17)	292 (28.00)
Decision making	[*p* = 0.260]	[*p* = 0.411]	[*p* = 0.553]	[*p* = 0.360]	[*p* = 0.351]	[*p* = 0.659]
Low empowerment	377 (21.26)	147 (21.27)	230 (21.26)	687 (38.70)	254 (36.76)	433 (39.94)
Medium empowerment	658 (19.41)	214 (18.77)	444 (19.73)	1252 (36.82)	382 (33.45)	870 (38.53)
High empowerment	1279 (19.73)	426 (19.39)	853 (19.90)	2471 (37.74)	769 (34.89)	1702 (39.52)
Fathers’ education	[*p* < 0.001]	[*p* < 0.001]	[*p* < 0.001]	[*p* < 0.001]	[*p* < 0.001]	[*p* < 0.001]
No education	491 (27.87)	164 (26.37)	327 (28.68)	869 (49.07)	279 (44.64)	590 (51.48)
Primary	911 (23.72)	287 (23.51)	624 (23.83)	1672 (43.41)	478 (39.05)	1194 (45.43)
Secondary	692 (17.94)	251 (18.01)	441 (17.90)	1331 (34.38)	439 (31.45)	892 (36.03)
Higher	220 (10.06)	85 (10.75)	135 (9.67)	538 (24.55)	209 (26.39)	329 (23.52)
Mothers’ occupation	[*p* = 0.01]	[*p* = 0.335]	[*p* < 0.001]	[*p* = 0.005]	[*p* = 0.218]	[*p* = 0.001]
Not working	1388 (19.12)	575 (19.92)	813 (18.60)	2676 (36.75)	1024 (35.38)	1652 (37.65)
Working	926 (21.10)	212 (18.58)	714 (21.98)	1734 (39.37)	381 (33.33)	1353 (41.49)
Fathers’ occupation	[*p* = 0.625]	[*p* = 0.062]	[*p* = 0.173]	[*p* = 0.196]	[*p* = 0.096]	[*p* = 0.826]
Not working	24 (18.18)	8 (10.96)	16 (27.12)	43 (32.33)	19 (25.68)	24 (40.68)
Working	2290 (19.89)	779 (10.70)	1511 (19.99)	4367 (37.80)	1386 (34.97)	2981 (39.28)
Wealth index	[*p* < 0.001]	[*p* < 0.001]	[*p* < 0.001]	[*p* < 0.001]	[*p* < 0.001]	[*p* < 0.001]
Poorest	715 (27.84)	255 (29.65)	460 (26.93)	1256 (48.80)	404 (46.92)	852 (49.74)
Poorer	551 (23.63)	178 (22.47)	373 (24.22)	1013 (43.29)	305 (38.41)	708 (45.80)
Middle	433 (19.91)	159 (19.78)	274 (19.99)	790 (36.29)	272 (33.79)	518 (37.76)
Richer	373 (16.20)	109 (13.90)	264 (17.38)	784 (33.90)	227 (28.92)	557 (36.45)
Richest	242 (10.67)	86 (10.91)	156 (10.53)	567 (24.85)	197 (24.87)	370 (24.83)
Accessing healthcare	[*p* < 0.001]	[*p* = 0.067]	[*p* < 0.001]	[*p* < 0.001]	[*p* = 0.170]	[*p* < 0.001]
Not big problem	804 (16.88)	291 (18.13)	513 (16.24)	1637 (34.20)	540 (33.54)	1097 (34.54)
Big problem	1510 (21.94)	496 (20.47)	1014 (22.74)	2773 (40.19)	865 (35.64)	1908 (42.66)
Body mass index	[*p* < 0.001]	[*p* < 0.001]	[*p* < 0.001]	[*p* < 0.001]	[*p* < 0.001]	[*p* < 0.001]
Underweight	499 (30.22)	153 (29.03)	346 (30.78)	836 (50.36)	240 (45.37)	596 (52.70)
Normal	1384 (20.37)	474 (20.99)	910 (20.07)	2668 (39.15)	830 (36.66)	1838 (40.39)
Overweight	380 (14.67)	146 (14.46)	234 (14.81)	771 (29.69)	291 (28.78)	480 (30.26)
Obese	51 (8.32)	14 (6.01)	37 (9.74)	135 (21.99)	44 (18.88)	91 (23.88)
Religion	[*p* = 0.517]	[*p* = 0.222]	[*p* = 0.942]	[*p* = 0.288]	[*p* = 0.775]	[*p* = 0.265]
Muslim	2121 (19.94)	728 (19.77)	1393 (20.03)	4043 (37.88)	1287 (34.87)	2756 (39.48)
Others	193 (19.09)	59 (17.05)	134 (20.15)	367 (36.19)	118 (34.10)	249 (37.28)
Place of residence	[*p* < 0.001]	[*p* = 0.003]	[*p* < 0.001]	[*p* < 0.001]	[*p* = 0.047]	[*p* < 0.001]
Urban	672 (17.35)	217 (16.85)	455 (17.60)	1320 (33.93)	422 (32.64)	898 (34.58)
Rural	1642 (21.12)	570 (20.80)	1072 (21.30)	3090 (39.64)	983 (35.82)	2107 (41.71)
Division	[*p* < 0.001]	[*p* < 0.001]	[*p* < 0.001]	[*p* < 0.001]	[*p* < 0.001]	[*p* < 0.001]
Barisal	253 (19.91)	95 (20.21)	158 (19.75)	483 (37.94)	170 (36.09)	313 (39.03)
Chittagong	376 (19.57)	139 (19.97)	237 (19.35)	724 (37.59)	238 (34.20)	486 (39.51)
Dhaka	258 (15.66)	83 (14.31)	175 (16.39)	529 (31.98)	173 (29.73)	356 (33.21)
Khulna	203 (16.41)	71 (16.51)	132 (16.36)	383 (30.81)	128 (29.56)	255 (31.48)
Mymensingh	322 (23.03)	108 (22.04)	214 (23.57)	588 (42.00)	194 (39.59)	394 (43.30)
Rajshahi	211 (17.79)	66 (16.88)	145 (18.24)	434 (36.26)	122 (31.12)	312 (38.76)
Rangpur	242 (18.21)	90 (19.96)	152 (17.31)	471 (35.36)	156 (34.59)	315 (35.75)
Sylhet	449 (27.08)	135 (25.96)	314 (27.59)	798 (48.04)	224 (42.91)	574 (50.40)
Survey round	[*p* = 0.517]	-	-	[*p* < 0.001]	-	-
BDHS 2017-18	1527 (20.04)	-	-	3005 (39.29)	-	-
BDHS 2022	787 (19.54)	-	-	1405 (34.80)	-	-

Note: Values represent count with percentages in parentheses. BDHS (Bangladesh Demographic and Health Survey).

**Table 2:** Association between maternal empowerment domains and multiple undernutrition status among children as estimated by multivariable logistic regression models in the analyzed sample.

Maternal Empowerment Domains	Pooled Sample		BDHS 2022		BDHS 2017-18	
	AOR ^α^ (95% CI)	*p*-Value	AOR ^β^ (95% CI)	*p*-Value	AOR ^β^ (95% CI)	*p*-Value
Attitude to violence						
Low empowerment	Ref.		Ref.		Ref.	
Medium empowerment	0.75 (0.55, 1.00)	0.054	0.77 (0.47, 1.26)	0.296	0.73 (0.50, 1.06)	0.104
High empowerment	0.70 (0.54, 0.90)	0.007	0.63 (0.41, 0.97)	0.034	0.72 (0.51, 1.00)	0.051
Social independence						
Low empowerment	Ref.		Ref.		Ref.	
Medium empowerment	0.81 (0.73, 0.91)	<0.001	0.73 (0.59, 0.89)	0.002	0.86 (0.75, 0.98)	0.024
High empowerment	0.82 (0.69, 0.98)	0.026	0.75 (0.57, 0.99)	0.041	0.85 (0.68, 1.07)	0.165
Decision making						
Low empowerment	Ref.		Ref.		Ref.	
Medium empowerment	0.92 (0.78, 1.08)	0.315	0.92 (0.69, 1.22)	0.549	0.93 (0.76, 1.14)	0.487
High empowerment	0.94 (0.81, 1.10)	0.425	0.94 (0.74, 1.22)	0.679	0.94 (0.77, 1.14)	0.544

^α^ Adjusted for sampling unit, sampling strata, sampling weight, fathers’ education, fathers’ occupation, mothers’ occupation, wealth index, maternal status of accessing healthcare, maternal body mass index, religion, place of residence, division, and year of survey round; ^β^ Adjusted for sampling unit, sampling strata, sampling weight, fathers’ education, fathers’ occupation, mothers’ occupation, wealth index, maternal status of accessing healthcare, maternal body mass index, religion, place of residence, and division; AOR (Adjusted Odds Ratio); BDHS (Bangladesh Demographic and Health Survey); Ref. (Reference).

**Table 3:** Association between maternal empowerment domains and any undernutrition status among children as identified by multivariable logistic regression models in the analyzed sample.

Maternal Empowerment Domains	Pooled Sample		BDHS 2022		BDHS 2017-18	
	AOR ^α^ (95% CI)	*p*-Value	AOR ^β^ (95% CI)	*p*-Value	AOR ^β^ (95% CI)	*p*-Value
Attitude to violence						
Low empowerment	Ref.	-	Ref.	-	Ref.	-
Medium empowerment	1.03 (0.80, 1.33)	0.826	1.01 (0.65, 1.58)	0.952	1.05 (0.76, 1.43)	0.780
High empowerment	0.93 (0.74, 1.17)	0.515	0.82 (0.55, 1.21)	0.313	0.99 (0.75, 1.32)	0.954
Social independence						
Low empowerment	Ref.	-	Ref.	-	Ref.	-
Medium empowerment	0.87 (0.79, 0.96)	0.007	0.77 (0.65, 0.90)	0.002	0.93 (0.83, 1.05)	0.243
High empowerment	0.82 (0.71, 0.95)	0.009	0.83 (0.67, 1.04)	0.108	0.78 (0.64, 0.95)	0.014
Decision making						
Low empowerment	Ref.	-	Ref.	-	Ref.	-
Medium empowerment	0.96 (0.83, 1.10)	0.541	0.93 (0.74, 1.17)	0.544	0.98 (0.82, 1.17)	0.830
High empowerment	1.02 (0.89, 1.16)	0.791	0.98 (0.80, 1.20)	0.831	1.07 (0.90, 1.26)	0.457

^α^ Adjusted for sampling unit, sampling strata, sampling weight, fathers’ education, fathers’ occupation, mothers’ occupation, wealth index, maternal status of accessing healthcare, maternal body mass index, religion, place of residence, division, and year of survey round; ^β^ Adjusted for sampling unit, sampling strata, sampling weight, fathers’ education, fathers’ occupation, mothers’ occupation, wealth index, maternal status of accessing healthcare, maternal body mass index, religion, place of residence, and division; AOR (Adjusted Odds Ratio); BDHS (Bangladesh Demographic and Health Survey); Ref. (Reference).

## Data Availability

Publicly accessible Bangladesh Demographic and Health (BDHS) data were used in this study. The data can be accessed either at https://dhsprogram.com/ (accessed on 6 September 2024) or upon reasonable request to the corresponding author. The full statistical models are presented as the Supplementary Materials.

## Supplementary Materials

The following supporting information can be downloaded at: https://www.mdpi.com/article/10.3390/nu18111730/s1. Table S1: STROBE checklist; Table S2: Items in SWPER index domains, their coding and cut-off points; Table S3: Association between maternal empowerment domains and stunting status among sampled children; Table S4: Association between maternal empowerment domains and wasting status among sampled children; Table S5: Association between maternal empowerment domains and underweight status among sampled children; Table S6: Association between attitude to violence domain of maternal empowerment and childhood multiple undernutrition based on multivariable logistic regression model; Table S7: Association between social independence domain of maternal empowerment and childhood multiple undernutrition based on multivariable logistic regression model; Table S8: Association between decision making domain of maternal empowerment and childhood multiple undernutrition based on multivariable logistic regression model; Table S9: Association between attitude to violence domain of maternal empowerment and childhood any undernutrition based on multivariable logistic regression model; Table S10: Association between social independence domain of maternal empowerment and childhood any undernutrition based on multivariable logistic regression model; Table S11: Association between decision making domain of maternal empowerment and childhood any undernutrition based on multivariable logistic regression model; Table S12: Association of childhood undernutrition with maternal age at delivery, education, and exposure to media.

## Abbreviations

The following abbreviations are used in this manuscript.

AOR	Adjusted Odds Ratio
BDHS	Bangladesh Demographic and Health Survey
CI	Confidence Interval
DHS	Demographic and Health Survey
HAZ	Height-for-Age Z-score
STROBE	Strengthening the Reporting of Observational studies in Epidemiology
SWPER	Survey-based Women’s Empowerment Index
UNICEF	United Nations Children’s Fund
VIF	Variance Inflation Factor
WAZ	Weight-for-Age Z-score
WHO	World Health Organization
WHZ	Weight-for-Height Z-score
