# Supplementary material for: Effects of Maternal Empowerment on Childhood Undernutrition in Bangladesh: Findings from Nationally Representative Surveys
Source: Nutrients. 2026 May 28;18(11):1730. doi: 10.3390/nu18111730 (PMC13259548; doi:10.3390/nu18111730)
Supplement: Supplementary file 1 [file nutrients-18-01730-s001.zip › nutrients-4321541-supplementary.pdf]

nutrients

Supplementary Material

Effects of Maternal Empowerment on Childhood Undernutrition in Bangladesh: Findings from Nationally Representative Surveys

Contents

Table S1: STROBE checklist 2

Table S2: Items in SWPER index domains, their coding and cut-off points 4

Table S3: Association between maternal empowerment domains and stunting status among sampled children 5

Table S4: Association between maternal empowerment domains and wasting status among sampled children 6

Table S5: Association between maternal empowerment domains and underweight status among sampled children 7

Table S6: Association between attitude to violence domain of maternal empowerment and childhood multiple undernutrition based on multivariable logistic regression model 8

Table S7: Association between social independence domain of maternal empowerment and childhood multiple undernutrition based on multivariable logistic regression model 11

Table S8: Association between decision making domain of maternal empowerment and childhood multiple undernutrition based on multivariable logistic regression model 14

Table S9: Association between attitude to violence domain of maternal empowerment and childhood any undernutrition based on multivariable logistic regression model 17

Table S10: Association between social independence domain of maternal empowerment and childhood any undernutrition based on multivariable logistic regression model 20

Table S11: Association between decision making domain of maternal empowerment and childhood any undernutrition based on multivariable logistic regression model 23

Table S12: Association of childhood undernutrition with maternal age at delivery, education, and exposure to media 26

nutrients

MDPI

Table S1: STROBE checklist

STROBE Statement—Checklist of items that should be included in reports of ***cross-sectional studies***

	Item No	Recommendation	Reporting location in the manuscript
**Title and abstract**	1	(*a*) Indicate the study's design with a commonly used term in the title or the abstract	Title
		(*b*) Provide in the abstract an informative and balanced summary of what was done and what was found	Abstract
**Introduction**			
Background/rationale	2	Explain the scientific background and rationale for the investigation being reported	Paragraph 4
Objectives	3	State specific objectives, including any prespecified hypotheses	Paragraph 4
**Methods**			
Study design	4	Present key elements of study design early in the paper	Study design and sample
Setting	5	Describe the setting, locations, and relevant dates, including periods of recruitment, exposure, follow-up, and data collection	Study design and sample
Participants	6	(*a*) Give the eligibility criteria, and the sources and methods of selection of participants	Study design and sample
Variables	7	Clearly define all outcomes, exposures, predictors, potential confounders, and effect modifiers. Give diagnostic criteria, if applicable	Exposure variables, Outcome variables, and Covariates
Data sources/ measurement	8*	For each variable of interest, give sources of data and details of methods of assessment (measurement). Describe comparability of assessment methods if there is more than one group	Study design and sample, Exposure variables, Outcome variables, and Covariates
Bias	9	Describe any efforts to address potential sources of bias	Statistical analysis
Study size	10	Explain how the study size was arrived at	Figure 1
Quantitative variables	11	Explain how quantitative variables were handled in the analyses. If applicable, describe which groupings were chosen and why	Statistical analysis
Statistical methods	12	(*a*) Describe all statistical methods, including those used to control for confounding	Statistical analysis
		(*b*) Describe any methods used to examine subgroups and interactions	Not applicable
		(*c*) Explain how missing data were addressed	Statistical analysis, Figure 1
		(*d*) If applicable, describe analytical methods taking account of sampling strategy	Study design and sample
		(*e*) Describe any sensitivity analyses	Statistical analysis

nutrients MDPI

**Results**			
Participants	13*	(a) Report numbers of individuals at each stage of study—eg numbers potentially eligible, examined for eligibility, confirmed eligible, included in the study, completing follow-up, and analyzed	Paragraph 1, Figure 1
		(b) Give reasons for non-participation at each stage	Figure 1
		(c) Consider use of a flow diagram	Figure 1
Descriptive data	14*	(a) Give characteristics of study participants (e.g., demographic, clinical, social) and information on exposures and potential confounders	Paragraph 1 and 2, Table 1
		(b) Indicate number of participants with missing data for each variable of interest	Figure 1
Outcome data	15*	Report numbers of outcome events or summary measures	Paragraph 2, Figure 2
Main results	16	(*a*) Give unadjusted estimates and, if applicable, confounder-adjusted estimates and their precision (e.g., 95% confidence interval). Make clear which confounders were adjusted for and why they were included	Table 2, 3
		(*b*) Report category boundaries when continuous variables were categorized	Not applicable
		(*c*) If relevant, consider translating estimates of relative risk into absolute risk for a meaningful time period	Not applicable
Other analyses	17	Report other analyses done—e.g., analyses of subgroups and interactions, and sensitivity analyses	Not applicable
**Discussion**			
Key results	18	Summarize key results with reference to study objectives	Paragraph 1
Limitations	19	Discuss limitations of the study, taking into account sources of potential bias or imprecision. Discuss both direction and magnitude of any potential bias	Paragraph 4
Interpretation	20	Give a cautious overall interpretation of results considering objectives, limitations, multiplicity of analyses, results from similar studies, and other relevant evidence	Paragraph 2
Generalizability	21	Discuss the generalizability (external validity) of the study results	Paragraph 3
**Other information**			
Funding	22	Give the source of funding and the role of the funders for the present study and, if applicable, for the original study on which the present article is based	Funding statement

*Give information separately for exposed and unexposed groups.

Note: An Explanation and Elaboration article discusses each checklist item and gives methodological background and published examples of transparent reporting. The STROBE checklist is best used in conjunction with this article (freely available on the Web sites of PLoS Medicine at

nutrients MDPI

http://www.plosmedicine.org/, Annals of Internal Medicine at http://www.annals.org/, and Epidemiology at http://www.epidem.com/). Information on the STROBE Initiative is available at www.strobe-statement.org.

Table S2: Items in SWPER index domains, their coding and cut-off points

Domains of SWPER (Chronbach's alpha for all items= 0.69)	DHS Questions or items	Code
**Attitude to violence (Chronbach's alpha= 0.68)**	**In your opinion, is a husband justified in hitting or beating his wife in the following situations:**	
1	If she goes out without telling him?	
2	If she neglects the children?	Justified= – 1; not justified= 1; don't know= 0
3	If she argues with him?	
4	If she refuses to have sex with him?	
5	If she burns the food?	
**Social independence (Chronbach's alpha= 0.71)**		
6	Frequency of reading newspaper or magazine	Not at all= 0; <once a week= 1; ≥once a week= 2
7	Woman education in completed years of schooling	Years
8	Age of woman at first birth*	Years
9	Age at first cohabitation	Years
10	Age difference: woman's age minus husband's age	Years
11	Education difference: woman's minus husband's years of schooling	Years
**Decision-making domain (Chronbach's alpha= 0.78)**		
12	Who usually decides on respondent's health care	
13	Who usually decides on large household purchases	Husband or other alone=-1; Joint decision or respondent alone=1
14	Who usually decides on visits to family or relatives	

Cut-off points used to categorize the SWPER domains into low, medium and high empowerment levels

	Attitude to violence	Social independence	Decision-making
Low empowerment	≤−0.700	≤−0.559	≤−1.000
Medium empowerment	>−0.700 ≤0.400	>−0.559 ≤0.293	>−1.000 ≤0.600
High empowerment	>0.400	>0.293	>0.600

nutrients

MDPI

Table S3: Association between maternal empowerment domains and stunting status among sampled children

Maternal empowerment domains	Pooled sample		BDHS 2022		BDHS 2017-18	
	AOR[α] (95% CI)	p-value	AOR[β] (95% CI)	p-value	AOR[β] (95% CI)	p-value
**Attitude to violence**						
Low empowerment	Ref		Ref		Ref	
Medium empowerment	1.02 (0.78, 1.34)	0.87	1.08 (0.68, 1.71)	0.76	1.01 (0.72, 1.40)	0.97
High empowerment	0.92 (0.72, 1.18)	0.52	0.91 (0.59, 1.40)	0.67	0.93 (0.69, 1.26)	0.66
**Social independence**						
Low empowerment	Ref		Ref		Ref	
Medium empowerment	0.85 (0.77, 0.94)	0.002	0.74 (0.61, 0.89)	0.001	0.91 (0.80, 1.03)	0.12
High empowerment	0.79 (0.67, 0.93)	0.004	0.80 (0.62, 1.03)	0.08	0.75 (0.61, 0.93)	0.01
**Decision making**						
Low empowerment	Ref		Ref		Ref	
Medium empowerment	0.94 (0.81, 1.10)	0.48	0.88 (0.68, 1.15)	0.36	0.98 (0.80, 1.19)	0.83
High empowerment	0.97 (0.84, 1.13)	0.72	0.89 (0.71, 1.12)	0.34	1.02 (0.84, 1.23)	0.84

[α]Adjusted for sampling unit, sampling strata, sampling weight, fathers' education, fathers' occupation, mothers' occupation, wealth index, maternal status of accessing healthcare, maternal body mass index, religion, place of residence, division, and year of survey round; [β]Adjusted for sampling unit, sampling strata, sampling weight, fathers' education, fathers' occupation, mothers' occupation, wealth index, maternal status of accessing healthcare, maternal body mass index, religion, place of residence, and division; AOR (Adjusted Odds Ratio); BDHS (Bangladesh Demographic and Health Survey)

Table S4: Association between maternal empowerment domains and wasting status among sampled children

Maternal empowerment domains	Pooled sample		BDHS 2022		BDHS 2017-18	
	AOR[α] (95% CI)	p-value	AOR[β] (95% CI)	p-value	AOR[β] (95% CI)	p-value
**Attitude to violence**						
Low empowerment	Ref		Ref		Ref	
Medium empowerment	0.94 (0.62, 1.43)	0.78	0.89 (0.46, 1.74)	0.74	0.96 (0.57, 1.62)	0.89
High empowerment	0.84 (0.58, 1.21)	0.34	0.78 (0.43, 1.40)	0.40	0.86 (0.54, 1.36)	0.51
**Social independence**						
Low empowerment	Ref		Ref		Ref	
Medium empowerment	0.91 (0.78, 1.06)	0.22	0.85 (0.66, 1.08)	0.19	0.94 (0.77, 1.15)	0.55
High empowerment	0.98 (0.78, 1.23)	0.86	1.06 (0.77, 1.47)	0.71	0.84 (0.60, 1.19)	0.33
**Decision making**						
Low empowerment	Ref		Ref		Ref	
Medium empowerment	0.94 (0.77, 1.17)	0.60	089 (0.64, 1.24)	0.49	1.01 (0.77, 1.33)	0.93
High empowerment	1.0 (0.82, 1.22)	0.99	0.92 (0.68, 1.24)	0.59	1.08 (0.81, 1.42)	0.60

[α]Adjusted for sampling unit, sampling strata, sampling weight, fathers' education, fathers' occupation, mothers' occupation, wealth index, maternal status of accessing healthcare, maternal body mass index, religion, place of residence, division, and year of survey round; [β]Adjusted for sampling unit, sampling strata, sampling weight, fathers' education, fathers' occupation, mothers' occupation, wealth index, maternal status of accessing healthcare, maternal body mass index, religion, place of residence, and division; AOR (Adjusted Odds Ratio); BDHS (Bangladesh Demographic and Health Survey)

nutrients

MDPI

Table S5: Association between maternal empowerment domains and underweight status among sampled children

Maternal empowerment domains	Pooled sample		BDHS 2022		BDHS 2017-18	
	AOR$^{\alpha}$ (95% CI)	p-value	AOR$^{\beta}$ (95% CI)	p-value	AOR$^{\beta}$ (95% CI)	p-value
**Attitude to violence**						
Low empowerment	Ref		Ref		Ref	
Medium empowerment	0.76 (0.57, 1.01)	0.06	0.78 (0.48, 1.26)	0.32	0.75 (0.52, 1.08)	0.12
High empowerment	0.72 (0.56, 0.94)	0.01	0.63 (0.41, 0.95)	0.03	0.77 (0.56, 1.07)	0.12
**Social independence**						
Low empowerment	Ref		Ref		Ref	
Medium empowerment	0.83 (0.75, 0.93)	0.001	0.78 (0.65, 0.94)	0.01	0.85 (0.75, 0.97)	0.02
High empowerment	0.84 (0.71, 0.99)	0.04	0.79 (0.62, 1.01)	0.06	0.86 (0.68, 1.08)	0.19
**Decision making**						
Low empowerment	Ref		Ref		Ref	
Medium empowerment	0.95 (0.81, 1.11)	0.53	0.95 (0.73, 1.25)	0.72	0.96 (0.79, 1.17)	0.66
High empowerment	0.99 (0.85, 1.14)	0.86	1.01 (0.80, 1.29)	0.92	0.98 (0.82, 1.18)	0.83

$^{\alpha}$Adjusted for sampling unit, sampling strata, sampling weight, fathers' education, fathers' occupation, mothers' occupation, wealth index, maternal status of accessing healthcare, maternal body mass index, religion, place of residence, division, and year of survey round; $^{\beta}$Adjusted for sampling unit, sampling strata, sampling weight, fathers' education, fathers' occupation, mothers' occupation, wealth index, maternal status of accessing healthcare, maternal body mass index, religion, place of residence, and division; AOR (Adjusted Odds Ratio); BDHS (Bangladesh Demographic and Health Survey)

nutrients

MDPI

Table S6: Association between attitude to violence domain of maternal empowerment and childhood multiple undernutrition based on multivariable logistic regression model

Variables	Pooled sample		BDHS 2022		BDHS 2017-18	
	AOR (95% CI)	p-value	AOR (95% CI)	p-value	AOR (95% CI)	p-value
**Attitude to violence**						
Low empowerment	Ref	-	Ref	-	Ref	-
Medium empowerment	0.75 (0.55, 1.00)	0.054	0.77 (0.47, 1.26)	0.296	0.73 (0.50, 1.07)	0.104
High empowerment	0.70 (0.54, 0.90)	0.007	0.63 (0.41, 0.97)	0.034	0.72 (0.51, 1.00)	0.051
**Husband's education**						
No education	Ref	-	Ref	-	Ref	-
Primary	0.79 (0.68, 0.91)	0.002	0.82 (0.64, 1.06)	0.137	0.77 (0.64, 0.92)	0.005
Secondary	0.71 (0.60, 0.84)	< 0.001	0.82 (0.62, 1.07)	0.147	0.66 (0.53, 0.82)	< 0.001
Higher	0.46 (0.37, 0.57)	< 0.001	0.52 (0.36, 0.76)	0.001	0.43 (0.33, 0.57)	< 0.001
**Occupation**						
Not working	Ref	-	Ref	-	Ref	-
Working	1.03 (0.91, 1.15)	0.674	0.88 (0.72, 1.09)	0.252	1.10 (0.96, 1.27)	0.164
**Husband's occupation**						
Not working	Ref	-	Ref	-	Ref	-
Working	1.09 (0.66, 1.78)	0.746	1.67 (0.73, 3.83)	0.229	0.70 (0.38, 1.31)	0.269
**Wealth index**						

Poorest	Ref	-	Ref	-	Ref	-
Poorer	0.87 (0.75, 1.00)	0.057	0.77 (0.60, 1.00)	0.049	0.92 (0.77, 1.10)	0.369
Middle	0.76 (0.64, 0.89)	0.001	0.67 (0.52, 0.87)	0.002	0.82 (0.66, 1.01)	0.072
Richer	0.65 (0.54, 0.78)	< 0.001	0.45 (0.34, 0.62)	< 0.001	0.77 (0.61, 0.97)	0.027
Richest	0.51 (0.40, 0.64)	< 0.001	0.48 (0.33, 0.69)	< 0.001	0.53 (0.40, 0.71)	< 0.001
**Accessing healthcare**						
Not big problem	Ref	-	Ref	-	Ref	-
Big problem	1.07 (0.96, 1.20)	0.227	0.93 (0.77, 1.13)	0.446	1.16 (1.00, 1.33)	0.041
**Body mass index**						
Underweight	Ref	-	Ref	-	Ref	-
Normal	0.67 (0.58, 0.76)	< 0.001	0.74 (0.58, 0.95)	0.016	0.64 (0.54, 0.76)	< 0.001
Overweight	0.55 (0.45, 0.66)	< 0.001	0.53 (0.39, 0.72)	< 0.001	0.57 (0.45, 0.71)	< 0.001
Obese	0.33 (0.23, 0.47)	< 0.001	0.23 (0.12, 0.44)	< 0.001	0.40 (0.26, 0.61)	< 0.001
**Religion**						
Muslim	Ref	-	Ref	-	Ref	-
Others	0.87 (0.72, 1.06)	0.159	0.77 (0.53, 1.11)	0.160	0.91 (0.73, 1.15)	0.457
**Place of residence**						
Urban	Ref	-	Ref	-	Ref	-
Rural	0.82 (0.71, 0.94)	0.006	0.84 (0.68, 1.04)	0.101	0.82 (0.68, 0.99)	0.035
**Division**						

nutrients

MDPI

Dhaka	Ref	-	Ref	-	Ref	-
Barisal	1.16 (0.90, 1.49)	0.267	1.37 (0.91, 2.07)	0.135	1.04 (0.76, 1.43)	0.800
Chittagong	1.20 (0.96, 1.50)	0.104	1.35 (0.95, 1.90)	0.092	1.16 (0.88, 1.55)	0.294
Khulna	1.03 (0.81, 1.30)	0.819	1.16 (0.81, 1.67)	0.425	0.97 (0.71, 1.32)	0.831
Mymensingh	1.19 (0.95, 1.49)	0.124	1.23 (0.88, 1.70)	0.222	1.18 (0.88, 1.58)	0.273
Rajshahi	1.02 (0.78, 1.32)	0.910	1.12 (0.76, 1.64)	0.571	1.00 (0.71, 1.39)	0.956
Rangpur	0.97 (0.76, 1.22)	0.774	1.18 (0.82, 1.70)	0.367	0.87 (0.64, 1.18)	0.363
Sylhet	1.67 (1.37, 2.10)	< 0.001	1.72 (1.23, 2.42)	0.002	1.71 (1.30, 2.26)	< 0.001
**Survey round**						
BDHS 2017-18	Ref	-				
BDHS 2022	1.00 (0.89, 1.14)	0.878				

nutrients

MDPI

Table S7: Association between social independence domain of maternal empowerment and childhood multiple undernutrition based on multivariable logistic regression model

	Pooled sample		BDHS 2022		BDHS 2017-18	
Variables	AOR (95% CI)	P-value	AOR (95% CI)	P-value	AOR (95% CI)	P-value
**Social independence**						
Low empowerment	Ref	-	Ref	-	Ref	-
Medium empowerment	0.81 (0.73, 0.91)	< 0.001	0.73 (0.59, 0.89)	0.002	0.86 (0.75, 0.98)	0.024
High empowerment	0.82 (0.69, 0.98)	0.026	0.75 (0.57, 0.99)	0.041	0.85 (0.68, 1.07)	0.165
**Husband's education**						
No education	Ref	-	Ref	-	Ref	-
Primary	0.79 (0.68, 0.91)	0.002	0.81 (0.63, 1.04)	0.103	0.77 (0.64, 0.93)	0.005
Secondary	0.71 (0.60, 0.84)	< 0.001	0.80 (0.61, 1.05)	0.110	0.66 (0.54, 0.82)	< 0.001
Higher	0.47 (0.37, 0.59)	< 0.001	0.53 (0.37, 0.77)	0.001	0.44 (0.33, 0.59)	< 0.001
**Occupation**						
Not working	Ref	-	Ref	-	Ref	-
Working	1.02 (0.91, 1.15)	0.709	0.88 (0.63, 1.04)	0.002	1.10 (0.96, 1.26)	0.184
**Husband's occupation**						
Not working	Ref	-	Ref	-	Ref	-
Working	1.08 (0.65, 1.77)	0.772	1.66 (0.73, 3.78)	0.230	0.69 (0.37, 1.30)	0.256
**Wealth index**						

Poorest	Ref	-	Ref	-	Ref	-
Poorer	0.88 (0.77, 1.02)	0.094	0.78 (0.60, 1.00)	0.053	0.94 (0.79, 1.12)	0.477
Middle	0.77 (0.66, 0.91)	0.002	0.69 (0.53, 0.88)	0.004	0.83 (0.67, 1.04)	0.099
Richer	0.66 (0.55, 0.79)	< 0.001	0.46 (0.34, 0.62)	< 0.001	0.78 (0.62, 0.99)	0.043
Richest	0.53 (0.42, 0.66)	< 0.001	0.49 (0.34, 0.71)	< 0.001	0.55 (0.41, 0.74)	< 0.001
**Accessing healthcare**						
Not big problem	Ref	-	Ref	-	Ref	-
Big problem	1.07 (0.96, 1.20)	0.223	0.93 (0.77, 1.13)	0.482	1.16 (1.00, 1.33)	0.043
**Body mass index**						
Underweight	Ref	-	Ref	-	Ref	-
Normal	0.67 (0.58, 0.77)	< 0.001	0.75 (0.59, 0.95)	0.018	0.65 (0.54, 0.77)	< 0.001
Overweight	0.55 (0.46, 0.66)	< 0.001	0.53 (0.39, 0.73)	< 0.001	0.58 (0.46, 0.72)	< 0.001
Obese	0.34 (0.23, 0.48)	< 0.001	0.23 (0.12, 0.45)	< 0.001	0.41 (0.27, 0.62)	< 0.001
**Religion**						
Muslim	Ref	-	Ref	-	Ref	-
Others	0.88 (0.72, 1.07)	0.205	0.77 (0.53, 1.11)	0.159	0.93 (0.74, 1.17)	0.541
**Place of residence**						
Urban	Ref	-	Ref	-	Ref	-
Rural	0.83 (0.72, 0.95)	0.008	0.83 (0.67, 1.03)	0.091	0.83 (0.69, 1.00)	0.046
**Division**						

Dhaka	Ref	-	Ref	-	Ref	-
Barisal	1.18 (0.91, 1.52)	0.203	1.39 (0.92, 2.10)	0.120	1.07 (0.78, 1.46)	0.689
Chittagong	1.22 (0.98, 1.52)	0.079	1.39 (0.98, 1.97)	0.066	1.17 (0.88, 1.56)	0.268
Khulna	1.03 (0.81, 1.30)	0.817	1.15 (0.80, 1.66)	0.446	0.97 (0.71, 1.32)	0.835
Mymensingh	1.19 (0.96, 1.49)	0.115	1.24 (0.89, 1.72)	0.202	1.18 (0.88, 1.58)	0.272
Rajshahi	1.02 (0.78, 1.32)	0.907	1.12 (0.76, 1.64)	0.563	0.99 (0.70, 1.39)	0.953
Rangpur	0.96 (0.76, 1.22)	0.745	1.17 (0.81,1.69)	0.394	0.87 (0.64, 1.17)	0.357
Sylhet	1.74 (1.40, 2.16)	< 0.001	1.80 (1.28, 2.54)	0.001	1.74 (1.32, 2.29)	< 0.001
**Survey round**						
BDHS 2017-18	Ref	-				
BDHS 2022	1.03 (0.91, 1.16)	0.625				

nutrients

MDPI

Table S8: Association between decision making domain of maternal empowerment and childhood multiple undernutrition based on multivariable logistic regression model

	Pooled sample		BDHS 2022		BDHS 2017-18	
Variables	AOR (95% CI)	P-value	AOR (95% CI)	P-value	AOR (95% CI)	P-value
**Decision making**						
Low empowerment	Ref	-	Ref	-	Ref	-
Medium empowerment	0.92 (0.78, 1.08)	0.315	0.92 (0.69, 1.22)	0.549	0.93 (0.76, 1.14)	0.487
High empowerment	0.94 (0.81, 1.10)	0.425	0.95 (0.74, 1.22)	0.679	0.94 (0.77, 1.14)	0.544
**Husband's education**						
No education	Ref	-	Ref	-	Ref	-
Primary	0.78 (0.67, 0.91)	0.001	0.82 (0.63, 1.05)	0.120	0.76 (0.63, 0.92)	0.004
Secondary	0.70 (0.59, 0.83)	< 0.001	0.81 (0.61, 1.06)	0.122	0.65 (0.53, 0.81)	< 0.001
Higher	0.45 (0.36, 0.56)	< 0.001	0.51 (0.35, 0.74)	< 0.001	0.43 (0.32, 0.56)	< 0.001
**Occupation**						
Not working	Ref	-	Ref	-	Ref	-
Working	1.03 (0.92, 1.15)	0.638	0.89 (0.72, 1.10)	0.293	1.10 (0.96, 1.27)	0.163
**Husband's occupation**						
Not working	Ref	-	Ref	-	Ref	-
Working	1.09 (0.66, 1.80)	0.727	1.70 (0.74, 3.91)	0.215	0.70 (0.37, 1.32)	0.269
**Wealth index**						

Poorest	Ref	-	Ref	-	Ref	-
Poorer	0.87 (0.75, 1.00)	0.053	0.77 (0.59, 0.99)	0.043	0.92 (0.77, 1.10)	0.356
Middle	0.75 (0.64, 0.89)	0.001	0.66 (0.51, 0.85)	0.001	0.81 (0.65, 1.01)	0.062
Richer	0.64 (0.53, 0.77)	< 0.001	0.45 (0.33, 0.60)	< 0.001	0.76 (0.60, 0.96)	0.023
Richest	0.50 (0.40, 0.63)	< 0.001	0.47 (0.33, 0.68)	< 0.001	0.53 (0.39, 0.71)	< 0.001
**Accessing healthcare**						
Not big problem	Ref	-	Ref	-	Ref	-
Big problem	1.08 (0.97, 1.21)	0.174	0.94 (0.78, 1.14)	0.542	1.66 (1.01, 1.34)	0.035
**Body mass index**						
Underweight	Ref	-	Ref	-	Ref	-
Normal	0.67 (0.58, 0.77)	< 0.001	0.75 (0.59, 0.95)	0.019	0.64 (0.54, 0.73)	< 0.001
Overweight	0.55 (0.45, 0.66)	< 0.001	0.53 (0.39, 0.73)	< 0.001	0.57 (0.45, 0.72)	< 0.001
Obese	0.33 (0.23, 0.47)	< 0.001	0.23 (0.12, 0.45)	< 0.001	0.40 (0.26, 0.62)	< 0.001
**Religion**						
Muslim	Ref	-	Ref	-	Ref	-
Others	0.86 (0.71, 1.05)	0.135	0.77 (0.53, 1.10)	0.154	0.91 (0.72, 1.14)	0.410
**Place of residence**						
Urban	Ref	-	Ref	-	Ref	-
Rural	0.82 (0.71, 0.95)	0.006	0.83 (0.67, 1.03)	0.093	0.82 (0.68, 0.99)	0.039
**Division**						

Dhaka	Ref	-	Ref	-	Ref	-
Barisal	1.16 (0.90, 1.49)	0.258	1.33 (0.88, 2.00)	0.178	1.06 (0.77, 1.45)	0.735
Chittagong	1.20 (0.96, 1.50)	0.116	1.32 (0.93, 1.88)	0.117	1.16 (0.87, 1.55)	0.303
Khulna	1.03 (0.81, 1.30)	0.839	1.14 (0.79, 1.64)	0.487	0.97 (0.71, 1.32)	0.835
Mymensingh	1.18 (0.94, 1.47)	0.150	1.19 (0.86, 1.66)	0.292	1.17 (0.87, 1.57)	0.291
Rajshahi	1.01 (0.78, 1.32)	0.914	1.11 (0.75, 1.62)	0.604	0.99 (0.71, 1.39)	0.960
Rangpur	0.96 (0.76, 1.21)	0.728	1.15 (0.79, 1.65)	0.465	0.87 (0.64, 1.18)	0.368
Sylhet	1.68 (1.35, 2.09)	< 0.001	1.68 (1.20, 2.37)	0.003	1.70 (1.29, 2.25)	< 0.001
**Survey round**						
BDHS 2017-18	Ref	-				
BDHS 2022	1.00 (0.89, 1.13)	0.945				

Table S9: Association between attitude to violence domain of maternal empowerment and childhood any undernutrition based on multivariable logistic regression model

	Pooled sample		BDHS 2022		BDHS 2017-18	
Variables	AOR (95% CI)	P-value	AOR (95% CI)	P-value	AOR (95% CI)	P-value
**Attitude to violence**						
Low empowerment	Ref	-	Ref	-	Ref	-
Medium empowerment	1.03 (0.80, 1.33)	0.826	1.01 (0.65, 1.58)	0.952	1.05 (0.76, 1.43)	0.780
High empowerment	0.93 (0.74, 1.17)	0.515	0.82 (0.55, 1.21)	0.313	0.99 (0.75, 1.32)	0.954
**Husband's education**						
No education	Ref	-	Ref	-	Ref	-
Primary	0.78 (0.69, 0.89)	< 0.001	0.78 (0.62, 0.98)	0.030	0.78 (0.67, 0.92)	0.003
Secondary	0.68 (0.59, 0.79)	< 0.001	0.73 (0.58, 0.92)	0.009	0.66 (0.55, 0.79)	< 0.001
Higher	0.51 (0.43, 0.61)	< 0.001	0.66 (0.50, 0.89)	0.006	0.45 (0.36, 0.56)	< 0.001
**Occupation**						
Not working	Ref	-	Ref	-	Ref	-
Working	1.01 (0.91, 1.12)	0.818	0.91 (0.76, 1.09)	0.284	1.06 (0.93, 1.20)	0.387
**Husband's occupation**						
Not working	Ref	-	Ref	-	Ref	-
Working	1.10 (0.73, 1.65)	0.656	1.15 (0.62, 2.13)	0.649	1.00 (0.59, 1.69)	0.985
**Wealth index**						

Poorest	Ref	-	Ref	-	Ref	-
Poorer	0.86 (0.75, 0.99)	0.032	0.80 (0.62, 1.03)	0.081	0.89 (0.76, 1.05)	0.171
Middle	0.70 (0.61, 0.81)	< 0.001	0.64 (0.51, 0.81)	< 0.001	0.74 (0.62, 0.88)	0.001
Richer	0.69 (0.60, 0.81)	< 0.001	0.53 (0.41, 0.70)	< 0.001	0.79 (0.66, 0.95)	0.011
Richest	0.56 (0.47, 0.68)	< 0.001	0.56 (0.41, 0.75)	< 0.001	0.57 (0.45, 0.72)	< 0.001
**Accessing healthcare**						
Not big problem	Ref	-	Ref	-	Ref	-
Big problem	1.01 (0.92, 1.11)	0.866	0.94 (0.80, 1.10)	0.431	1.05 (0.93, 1.18)	0.412
**Body mass index**						
Underweight	Ref	-	Ref	-	Ref	-
Normal	0.71 (0.62, 0.81)	< 0.001	0.76 (0.61, 0.95)	0.016	0.68 (0.58, 0.80)	< 0.001
Overweight	0.56 (0.48, 0.65)	< 0.001	0.57 (0.44, 0.75)	< 0.001	0.55 (0.45, 0.66)	< 0.001
Obese	0.44 (0.34, 0.56)	< 0.001	0.37 (0.24, 0.58)	< 0.001	0.48 (0.35, 0.65)	< 0.001
**Religion**						
Muslim	Ref	-	Ref	-	Ref	-
Others	0.87 (0.74, 1.01)	0.070	0.93 (0.71, 1.21)	0.582	0.84 (0.69, 1.01)	0.069
**Place of residence**						
Urban	Ref	-	Ref	-	Ref	-
Rural	0.88 (0.78, 1.00)	0.059	0.79 (0.65, 0.97)	0.022	0.95 (0.80, 1.12)	0.507
**Division**						

Dhaka	Ref	-	Ref	-	Ref	-
Barisal	1.16 (0.95, 1.41)	0.147	1.23 (0.89, 1.68)	0.205	1.12 (0.87, 1.45)	0.386
Chittagong	1.20 (0.99, 1.44)	0.065	1.14 (0.84, 1.53)	0.402	1.25 (0.97, 1.59)	0.079
Khulna	0.91 (0.75, 1.12)	0.375	0.99 (0.74, 1.34)	0.968	0.88 (0.67, 1.14)	0.328
Mymensingh	1.16 (0.95, 1.40)	0.137	1.22 (0.93, 1.61)	0.153	1.13 (0.87, 1.46)	0.359
Rajshahi	1.09 (0.89, 1.33)	0.429	0.99 (0.72, 1.36)	0.933	1.14 (0.88, 1.47)	0.338
Rangpur	0.95 (0.79, 1.16)	0.637	1.07 (0.80, 1.44)	0.644	0.90 (0.70, 1.16)	0.421
Sylhet	1.64 (1.37, 1.97)	< 0.001	1.52 (1.15, 2.01)	0.003	1.73 (1.37, 2.18)	< 0.001
**Survey round**						
BDHS 2017-18	Ref	-				
BDHS 2022	0.85 (0.76, 0.94)	0.003				

nutrients

MDPI

Table S10: Association between social independence domain of maternal empowerment and childhood any undernutrition based on multivariable logistic regression model

	Pooled sample		BDHS 2022		BDHS 2017-18	
Variables	AOR (95% CI)	P-value	AOR (95% CI)	P-value	AOR (95% CI)	P-value
**Social independence**						
Low empowerment	Ref	-	Ref	-	Ref	-
Medium empowerment	0.87 (0.79, 0.96)	0.007	0.77 (0.65, 0.90)	0.002	0.93 (0.83, 1.05)	0.243
High empowerment	0.82 (0.71, 0.95)	0.009	0.83 (0.67, 1.04)	0.108	0.78 (0.64, 0.95)	0.014
**Husband's education**						
No education	Ref	-	Ref	-	Ref	-
Primary	0.78 (0.69, 0.89)	< 0.001	0.77 (0.61, 0.96)	0.021	0.79 (0.67, 0.92)	0.003
Secondary	0.69 (0.60, 0.79)	< 0.001	0.72 (0.57, 0.91)	0.006	0.67 (0.55, 0.80)	< 0.001
Higher	0.53 (0.44, 0.63)	< 0.001	0.67 (0.50, 0.92)	0.008	0.47 (0.37, 0.58)	< 0.001
**Occupation**						
Not working	Ref	-	Ref	-	Ref	-
Working	1.01 (0.91, 1.12)	0.820	0.91 (0.77, 1.08)	0.281	1.06 (0.93, 1.20)	0.365
**Husband's occupation**						
Not working	Ref	-	Ref	-	Ref	-
Working	1.09 (0.73, 1.64)	0.670	1.16 (0.63, 2.13)	0.626	0.99 (0.59, 1.69)	0.983
**Wealth index**						

Poorest	Ref	-	Ref	-	Ref	-
Poorer	0.87 (0.76, 1.00)	0.049	0.80 (0.62, 1.03)	0.083	0.90 (0.77,1.06)	0.211
Middle	0.72 (0.62, 0.83)	< 0.001	0.65 (0.52, 0.83)	< 0.001	0.75 (0.63, 0.90)	0.002
Richer	0.71 (0.61, 0.82)	< 0.001	0.53 (0.41, 0.70)	< 0.001	0.81 (0.68, 0.97)	0.023
Richest	0.58 (0.48, 0.70)	< 0.001	0.56 (0.42, 0.76)	< 0.001	0.60 (0.47, 0.76)	< 0.001
**Accessing healthcare**						
Not big problem	Ref	-	Ref	-	Ref	-
Big problem	1.01 (0.92, 1.10)	0.889	0.94 (0.80, 1.10)	0.442	1.05 (0.93, 1.17)	0.449
**Body mass index**						
Underweight	Ref	-	Ref	-	Ref	-
Normal	0.71 (0.62, 0.81)	< 0.001	0.77 (0.62, 0.95)	0.016	0.68 (0.58, 0.80)	< 0.001
Overweight	0.56 (0.48, 0.65)	< 0.001	0.58 (0.44, 0.75)	< 0.001	0.55 (0.46, 0.67)	< 0.001
Obese	0.44 (0.35, 0.57)	< 0.001	0.38 (0.24, 0.59)	< 0.001	0.48 (0.35, 0.66)	< 0.001
**Religion**						
Muslim	Ref	-	Ref	-	Ref	-
Others	0.88 (0.75, 1.03)	0.109	0.93 (0.71, 1.21)	0.573	0.86 (0.71, 1.04)	0.125
**Place of residence**						
Urban	Ref	-	Ref	-	Ref	-
Rural	0.89 (0.78, 1.01)	0.065	0.79 (0.65, 0.96)	0.021	0.95 (0.80, 1.12)	0.508
**Division**						

Dhaka	Ref	-	Ref	-	Ref	-
Barisal	1.17 (0.96, 1.43)	0.110	1.24 (0.91, 1.71)	0.177	1.13 (0.88, 1.45)	0.347
Chittagong	1.21 (1.00, 1.46)	0.049	1.16 (0.86, 1.57)	0.322	1.25 (0.98, 1.60)	0.070
Khulna	0.91 (0.75, 1.11)	0.369	0.99 (0.73, 1.33)	0.933	0.88 (0.67, 1.14)	0.318
Mymensingh	1.16 (0.96, 1.41)	0.126	1.23 (0.93, 1.63)	0.139	1.13 (0.88, 1.46)	0.342
Rajshahi	1.09 (0.89, 1.33)	0.426	0.99 (0.72, 1.36)	0.948	1.13 (0.88, 1.47)	0.338
Rangpur	0.95 (0.79, 1.15)	0.615	1.07 (0.79, 1.43)	0.668	0.90 (0.70, 1.15)	0.403
Sylhet	1.68 (1.40, 2.01)	< 0.001	1.57 (1.18, 2.08)	0.002	1.76 (1.40, 2.22)	< 0.001
**Survey round**						
BDHS 2017-18	Ref	-				
BDHS 2022	0.86 (0.77, 0.96)	0.006				

Table S11: Association between decision making domain of maternal empowerment and childhood any undernutrition based on multivariable logistic regression model

	Pooled sample		BDHS 2022		BDHS 2017-18	
Variables	AOR (95% CI)	P-value	AOR (95% CI)	P-value	AOR (95% CI)	P-value
**Decision making**						
Low empowerment	Ref	-	Ref	-	Ref	-
Medium empowerment	0.96 (0.83, 1.10)	0.568	0.93 (0.74, 1.17)	0.544	0.98 (0.82, 1.17)	0.830
High empowerment	1.03 (0.90, 1.17)	0.673	0.98 (0.80, 1.20)	0.831	1.07 (0.90, 1.26)	0.457
**Husband's education**						
No education	Ref	-	Ref	-	Ref	-
Primary	0.78 (0.69, 0.89)	< 0.001	0.77 (0.62, 0.97)	0.025	0.79 (0.67, 0.92)	0.003
Secondary	0.69 (0.59, 0.79)	< 0.001	0.73 (0.58, 0.92)	0.007	0.66 (0.55, 0.80)	< 0.001
Higher	0.51 (0.43, 0.61)	< 0.001	0.65 (0.49, 0.87)	0.004	0.45 (0.36, 0.56)	< 0.001
**Occupation**						
Not working	Ref	-	Ref	-	Ref	-
Working	1.01 (0.91, 1.12)	0.861	0.91 (0.77, 1.09)	0.309	1.05 (0.92, 1.19)	0.451
**Husband's occupation**						
Not working	Ref	-	Ref	-	Ref	-
Working	1.11 (0.73, 1.67)	0.627	1.17 (0.63, 2.16)	0.624	1.00 (0.59, 1.71)	0.988
**Wealth index**						

Poorest	Ref	-	Ref	-	Ref	-
Poorer	0.86 (0.75, 0.99)	0.034	0.79 (0.62, 1.02)	0.075	0.90 (0.76, 1.05)	0.185
Middle	0.71 (0.61, 0.81)	< 0.001	0.64 (0.51, 0.80)	< 0.001	0.74 (0.62, 0.89)	0.001
Richer	0.69 (0.60, 0.81)	< 0.001	0.53 (0.40, 0.69)	< 0.001	0.79 (0.66, 0.95)	0.013
Richest	0.56 (0.47, 0.68)	< 0.001	0.55 (0.41, 0.74)	< 0.001	0.57 (0.45, 0.73)	< 0.001
**Accessing healthcare**						
Not big problem	Ref	-	Ref	-	Ref	-
Big problem	1.01 (0.92, 1.11)	0.760	0.94 (0.81, 1.11)	0.485	1.06 (0.94, 1.19)	0.350
**Body mass index**						
Underweight	Ref	-	Ref	-	Ref	-
Normal	0.71 (0.62, 0.80)	< 0.001	0.77 (0.62, 0.95)	0.017	0.68 (0.58, 0.80)	< 0.001
Overweight	0.55 (0.48, 0.65)	< 0.001	0.58 (0.44, 0.75)	< 0.001	0.55 (0.45, 0.66)	< 0.001
Obese	0.43 (0.34, 0.56)	< 0.001	0.37 (0.24, 0.58)	< 0.001	0.47 (0.35, 0.65)	< 0.001
**Religion**						
Muslim	Ref	-	Ref	-	Ref	-
Others	0.86 (0.74, 1.01)	0.061	0.93 (0.71, 1.21)	0.573	0.84 (0.69, 1.01)	0.062
**Place of residence**						
Urban	Ref	-	Ref	-	Ref	-
Rural	0.89 (0.78, 1.01)	0.072	0.79 (0.65, 0.97)	0.022	0.95 (0.81, 1.13)	0.572
**Division**						

Dhaka	Ref	-	Ref	-	Ref	-
Barisal	1.16 (0.95, 1.42)	0.136	1.21 (0.88, 1.65)	0.248	1.13 (0.87, 1.45)	0.359
Chittagong	1.19 (0.98, 1.44)	0.072	1.12 (0.83, 1.51)	0.467	1.24 (0.97, 1.59)	0.084
Khulna	0.91 (0.75, 1.11)	0.370	0.98 (0.73, 1.32)	0.882	0.88 (0.68, 1.14)	0.337
Mymensingh	1.15 (0.94, 1.39)	0.169	1.19 (0.90, 1.58)	0.212	1.12 (0.87, 1.45)	0.390
Rajshahi	1.08 (0.88, 1.33)	0.436	0.98 (0.71, 1.34)	0.887	1.14 (0.88, 1.47)	0.332
Rangpur	0.95 (0.78,1.15)	0.598	1.05 (0.78, 1.41)	0.764	0.90 (0.70, 1.16)	0.424
Sylhet	1.64 (1.37, 1.97)	< 0.001	1.49 (1.12, 1.98)	0.006	1.74 (1.37, 2.19)	< 0.001
**Survey round**						
BDHS 2017-18	Ref	-				
BDHS 2022	0.84 (0.76, 0.94)	0.002				

nutrients

MDPI

Table S12: Association of childhood undernutrition with maternal age at delivery, education, and exposure to media

Maternal characteristics	Any undernutrition		Multiple undernutrition	
	OR (95% CI)	p-value	OR (95% CI)	p-value
**Maternal age at delivery**				
< 19 years	1	-	1	-
19-30 years	0.93 (0.84, 1.03)	0.17	0.94 (0.83, 1.07)	0.36
> 31 years	1.03 (0.91, 1.19)	0.57	1.11 (0.95, 1.31)	0.20
**Maternal education**				
No education	1	-	1	-
Primary	0.67 (0.57, 0.78)	< 0.001	0.59 (0.50, 0.69)	< 0.001
Secondary	0.46 (0.39, 0.53)	< 0.001	0.40 (0.34, 0.47)	< 0.001
Higher	0.23 (0.20, 0.28)	< 0.001	0.19 (0.15, 0.23)	< 0.001
**Exposure to media (TV, newspaper, radio)**				
No	1	-	1	-
Yes	0.76 (0.72, 0.81)	< 0.001	0.66 (0.60, 0.72)	< 0.001

Note: the association was estimated from pooled sample
